# Characteristic cardiac phenotypes are detected by cardiovascular magnetic resonance in patients with different clinical phenotypes and genotypes of mitochondrial myopathy

**DOI:** 10.1186/s12968-015-0145-x

**Published:** 2015-05-22

**Authors:** Anca Florian, Anna Ludwig, Bianca Stubbe-Dräger, Matthias Boentert, Peter Young, Johannes Waltenberger, Sabine Rösch, Udo Sechtem, Ali Yilmaz

**Affiliations:** Department of Cardiology and Angiology, University Hospital Münster, Albert-Schweitzer-Campus 1, building A1, 48149 Münster, Germany; Division of Cardiology, Robert-Bosch-Krankenhaus, Stuttgart, Germany; Department of Sleep Medicine and Neuromuscular Disorders, University Hospital Münster, Münster, Germany

**Keywords:** Mitochondrial myopathy, Cardiomyopathy, Cardiovascular magnetic resonance, MELAS, CPEO

## Abstract

**Background:**

Mitochondrial myopathies (MM) are a heterogeneous group of inherited conditions resulting from a primary defect in the mitochondrial respiratory chain with consecutively impaired cellular energy metabolism. Small sized studies using mainly electrocardiography (ECG) and echocardiography have revealed cardiac abnormalities ranging from conduction abnormalities and arrhythmias to hypertrophic or dilated cardiomyopathy in these patients. Recently, characteristic patterns of cardiac involvement were documented by cardiovascular magnetic resonance (CMR) in patients with **c**hronic **p**rogressive **e**xternal **o**phthalmoplegia (CPEO)/**K**earns-**S**ayre **s**yndrome (KSS) and with **m**itochondrial **e**ncephalopathy with **l**actic **a**cidosis and **s**troke-like episodes (MELAS). The present study aimed to characterize the prevalence and pattern of cardiac abnormalities and to test the additional diagnostic value of CMR in this patient population. The hypothesis that different neuromuscular MM syndromes present with different cardiac disease phenotypes was evaluated.

**Methods:**

Sixty-four MM patients (50 ± 15 years, 44 % male) and 25 matched controls (52 ± 14 years, 36 % male) prospectively underwent cardiac evaluations including CMR (comprising cine- and late-gadolinium-enhancement (LGE) imaging). Based on the neuromuscular phenotype and genotype, the patients were grouped: a) CPEO/KSS (N = 33); b) MELAS/–like (N = 11); c) **m**yoclonic **e**pilepsy with **r**agged-**r**ed **f**ibers (MERRF) (N = 3) and d) other non-specific MM forms (N = 17).

**Results:**

Among the 64 MM patients, 34 (53 %) had at least one abnormal CMR finding: 18 (28 %) demonstrated an impaired left ventricular ejection-fraction (LV-EF <60 %), 14 (22 %) had unexplained LV hypertrophy and 21 (33 %) were LGE-positive. Compared to controls, MM patients showed significantly higher maximal wall thickness (10 ± 3 vs. 8 ± 2 mm, p = 0.005) and concentricity (LV mass to end-diastolic volume: 0.84 ± 0.27 vs. 0.67 ± 0.11, p < 0.0001) with frequent presence of non-ischemic LGE (30 % vs. 0 %, p = 0.001). CPEO/KSS showed a predominantly intramural pattern of LGE mostly confined to the basal LV inferolateral wall (8/10; 80 %) in addition to a tendency toward concentric remodelling. MELAS/-like patients showed the highest frequency of cardiac disease (in 10/11 (91 %)), a mostly concentric LV hypertrophy (6/9; 67 %) with or without LV systolic dysfunction and a predominantly focal, patchy LGE equally distributed among LV segments (8/11; 73 %). Patients with MERRF and non-specific MM had no particular findings. Pathological CMR findings indicating cardiac involvement were detected significantly more often than pathological ECG results or elevated cardiac serum biomarkers (34 (53 %) vs. 18 (28 %) vs. 21 (33 %); p = 0.008).

**Conclusion:**

Cardiac involvement is a frequent finding in MM patients – and particularly present in KSS/CPEO as well as MELAS/-like patients. Despite a high variability in clinical presentation, CPEO/KSS patients typically show an intramural pattern of LGE in the basal inferolateral wall whereas MELAS patients are characterized by overt concentric hypertrophy and a rather unique, focally accentuated and diffusely distributed LGE.

**Electronic supplementary material:**

The online version of this article (doi:10.1186/s12968-015-0145-x) contains supplementary material, which is available to authorized users.

## Background

Mitochondrial myopathies (MM) are a heterogeneous group of inherited conditions resulting from a primary defect in the mitochondrial respiratory chain with consecutively impaired cellular energy metabolism affecting multiple organ systems. Despite the high variability in clinical presentation and the poor genotype-phenotype correlation, several syndromes with characteristic symptoms have been defined [1]. These disorders have a progressive course associated with different degrees of neurological disability and in some instances with premature death primarily due to cardiac and neurological adverse events [2–5].

The overall prevalence of MM-related cardiomyopathy is difficult to estimate and varies according to syndrome and to the diagnostic approach used [6, 7]. Cardiac abnormalities ranging from preexcitation, conduction blocks and arrhythmias to dilated or hypertrophic cardiomyopathy phenotypes have been described in several syndromes like **c**hronic **p**rogressive **e**xternal **o**phthalmoplegia (CPEO), **K**earns-**S**ayre **s**yndrome (KSS), **m**itochondrial **e**ncephalopathy with **l**actic **a**cidosis and **s**troke-like episodes (MELAS), **m**yoclonic **e**pilepsy with **r**agged-**r**ed **f**ibers (MERRF) and Leigh syndrome. These data derive from case reports and small sized studies using mainly electrocardiography (ECG) and echocardiography for cardiac evaluation [2, 4, 5, 8–12].

Cardiovascular magnetic resonance (CMR) is a highly sensitive tool for depicting myocardial abnormalities in MM patients including tissue damage by late gadolinium enhancement (LGE) [13–16]. Recently, two small sized CMR studies suggested that characteristic patterns of cardiac involvement might be present in some of the above syndromes [17, 18]. As no disease modifying therapy exists, an early diagnosis of cardiac involvement – particularly by CMR studies - would permit a timely initiation of appropriate treatment strategies with potential improvement in patient prognosis [19].

The present study aimed to characterize the prevalence and pattern of cardiac abnormalities in a group of MM patients with different clinical phenotypes and to test the additional diagnostic value of CMR in this patient population. Moreover, the hypothesis that different neuromuscular MM syndromes present with different cardiac disease phenotypes was evaluated.

## Methods

### Study population

Sixty-four patients with known MM were prospectively enrolled between 2009 and 2014 as participants of the “Mito-HERZ” study that was already described elsewhere [18]. All patients underwent cardiac and neurological evaluations including multi-parametric CMR. The clinical diagnosis of MM had been previously confirmed based on molecular genetic testing and/or skeletal biopsy with appropriate findings in all patients [10]. Exclusion criteria were presence of claustrophobia and contraindications to CMR or to gadolinium contrast administration.

Based on the clinical (neuromuscular) phenotype and genetic findings, the patients were further grouped as follows: (1) CPEO/KSS (N = 33; 29 with CPEO and 4 with KSS); (2) MELAS and MELAS–like (N = 11; 7 with MELAS and 4 with MELAS-like); (3) MERRF (N = 3) and (4) other non-specific MM forms (N = 17) (see also Additional file 1). In addition, 25 healthy individuals matched for age, gender and cardiovascular risk factors with no history of cardiac disease were enrolled between March 2011 and May 2014 and represented the control group. Approval of the study protocol was obtained from the local ethics committee, and all participating patients provided written informed consent.

### Cardiac evaluation

Both MM patients and controls underwent cardiac work-up including a thorough clinical history, physical examination, 12-lead ECG and CMR. An ECG was considered abnormal whenever at least one of the following findings was present: (1) arrhythmia; (2) conduction abnormalities; (3) isolated ST-segment depression in two or more contiguous leads; (4) isolated inverted T-waves; (5) pathologic Q-waves; (6) a Sokolow-Lyon index > 35 mm as sign of LV hypertrophy. In the MM patients, blood samples were taken for laboratory analysis including total creatine kinase (CK) and the cardiac biomarkers troponin T (TnT) and brain natriuretic-peptide (NT-proBNP).

### CMR imaging protocol

ECG-gated CMR studies were performed on 1.5-T scanners (Aera, Siemens Medical Solutions, Erlangen, Germany and Achieva, Philips, Best, The Netherlands) using commercially available cardiac software, electrocardiographic triggering, and cardiac-dedicated surface coils. Cine-imaging was performed using a steady-state-free-precession (SSFP) sequence in three long-axis slices (four-, three- and two-chamber) and a stack of short-axis slices completely covering the LV. LGE-imaging was performed using a T1-weighted inversion recovery gradient-echo sequence 10-15 min after intravenous contrast administration (0.15 mmol/kg Magnevist®) in the same imaging planes as the cine-images.

### CMR image analysis

CMR analysis was performed off-line by two experienced readers. Ventricular volumes, ejection fraction and LV mass were derived by contouring endo- and epicardial borders on the short-axis cine images and indexed to body surface area. The papillary muscles were included in the LV cavity. LV hypertrophy was considered present whenever maximal end diastolic wall thickness was ≥ 13 mm in men and ≥ 12 mm in women. The ratio of LV mass to end-diastolic volume was used as an index of concentric hypertrophy [20]. LGE presence and pattern were visually assessed on the short-and long-axis images by using the AHA 17-segment model. LGE pattern was globally assessed as: ischemic (subendocardial and/or transmural) and non-ischemic (subepicardial and/or intramural). An abnormal CMR study was defined by: (1) a LV ejection fraction (EF) less than 60 % and/or (2) the presence of unexplained LV hypertrophy and/or (3) LGE presence in at least one myocardial segment.

### Statistical analysis

Continuous variables with normal distribution are expressed as mean ± SD. Categorical variables are expressed as frequency with percentage. Student’s t-test was used for comparison of normally distributed characteristics between MM patients and controls. Levene’s test was used for testing equality of variances. One-way ANOVA with Bonferroni post hoc correction was used for subgroup multiple comparison analyses. Dunnett’s post hoc test was used in the case of inequality of variances. The chi-square test with Yate’s correction was used to compare non-continuous variables expressed as proportions. Statistical analysis was performed using SPSS software for Windows (Version 19.0, IBM Corp., Armonk., NY). A p-value ≤ 0.05 was considered statistically significant.

## Results

### Patient characteristics

Demographic an main clinical characteristics of both patients and controls are shown in Table 1. Mean age was 50 ± 15 years for MM patients and 47 ± 12 years for controls. With regard to age, there was no significant difference between MM subgroups (p = 0.46).

**Table 1:** Patient general characteristics

	Total MM Patients (N = 64)	CPEO/KSS (N = 33)	MELAS/-like (N = 11)	MERRF (N = 3)	Other MM (N = 17)	Controls (N = 25)	p value
Age, yrs	50 ± 15	52 ± 14	44 ± 17	46 ± 3	50 ± 15	47 ± 12	0.45
Male, n (%)	28 (44)	12 (36)	3 (27)	2 (67)	11 (65)	12 (48)	0.22
Diabetes, n (%)	10 (16)	5 (15)	3 (27)	0 (0)	2 (12)	3 (12)	0.51
Hypertension, n (%)	10 (16)	6 (18)	0 (0)	0 (0)	4 (24)	5 (20)	0.79
MM family history, n (%)	17 (26)	4 (13)*	9 (80)	2 (67)	2 (13)*	-	**<0.001**
Previous cardiac diagnosis, n (%)	20 (31)	8 (24)	7 (64)	1 (33)	4 (24)	-	0.09
BMI, kg/m^2^	24 ± 4	23 ± 4	22 ± 4	25 ± 3	25 ± 4	26 ± 4	0.07
Lab results							
Elevated CK, n (%)	29 (45)	14 (42)	5 (46)	2 (67)	8 (47)	-	0.89
Elevated TnT, n (%)	8 (13)	1 (3)*	5 (46)	1 (33)	1 (6)*	-	**0.003**
Elevated NT-proBNP, n (%)	16 (25)	10 (30)	4 (36)	0 (0)	2 (11)	-	0.29
Any elevated cardiac biomarkers, n (%)	21 (33)	11 (33)	6 (55)	1 (33)	3 (18)	-	0.23
ECG findings							
Sinus rhythm, n (%)	64 (100)	33 (100)	11 (100)	3 (100)	17 (100)	25 (100)	1.00
QRS abnormalities, n (%)	11 (17)	5 (15)	5 (46)§	1 (33)	0 (0)*	2 (8)	0.01
ST/T abnormalities, n (%)	11 (17)	3 (9)*	8 (73)§	0 (0)	0 (0)*	2 (8)	**<0.001**
Any abnormal ECG findings, n (%)	18 (28)	8 (24)*	9 (82)§	1 (33)*	0 (0)*	4 (16)	**<0.001**
Medication							
ACE inhibitor/ARB, n (%)	16 (25)	9 (27)	3 (20)	0 (0)	5 (29)	5 (20)	0.89
Beta blocker, n (%)	9 (14)	5 (15)	1 (10)	0 (0)	3 (18)	2 (8)	0.90
CoQ10, n (%)	23 (36)	10 (30)	6 (55)	1 (33)	6 (35)	-	0.51
Creatin/Carnitin, n (%)	9 (14)	7 (21)	0 (0)	1 (33)	1 (6)	-	0.16
Vitamins, n (%)	15 (24)	9 (27)	0 (0)	1 (33)	5 (31)	-	0.15

MM – mithochondrial myopathy; BMI – body mass index; CK – creatine kinase; TnT – troponin T; ACE – angiotensin converting enzyme; ARB – angiotensin receptor blocker; CoQ10 – coenzyme Q10

*- post Hoc p < 0.05 vs. MELAS/-like; § - vs. Control

Bold data indicates either significant or most important results

Twenty-six percent (N = 17) of the patients had a positive family history for MM. MELAS/-like patients presented significantly more often with a positive family history compared to CPEO/KSS and other MM patients. Thirty-one percent of MM patients (N = 20) had previously received a cardiac diagnosis as follows: arrhythmia (N = 8), impaired LV systolic function (N = 5), LV hypertrophy (N = 3), coronary artery disease (other than infarct) (N = 3) and left bundle branch block (N = 1). Although MELAS/-like patients had an almost double rate of previous heart disease diagnosis compared to the other MM subgroups, this difference was not statistically significant.

Table 2 summarizes the frequencies of different clinical features in MM patients. Nineteen percent of patients (N = 12) presented with chest pain symptoms and 47 % (N = 30) with exertional dyspnoea. Altogether, 48 % of MM patients had symptoms of possible cardiac origin at inclusion. No significant difference between MM groups regarding cardiac symptoms frequency was noted (p = 0.06 for any possible cardiac symptoms). Interestingly, MELAS/-like patients were the least symptomatic subgroup.

**Table 2:** Frequency of clinical features

N (%)	Total MM Patients (N = 64)	CPEO/KSS (N = 33)	MELAS/-like (N = 11)	MERRF (N = 3)	Other MM (N = 17)
Chest pain symptoms	12 (19)	5 (15)	1 (10)	1 (33)	5 (29)
Exercise dyspnea	30 (47)	17 (52)	2 (18)	3 (100)	8 (47)
Any cardiac symptom	31 (48)	17 (52)	2 (18)	3 (100)	9 (53)
Skeletal myopathy	44 (69)	23 (70)	9 (82)	2 (67)	10 (59)
Encephalopathy	5 (8)	0 (0)	4 (36)	0 (0)	1 (6)
Seizures	5 (8)	0 (0)	3 (27)	1 (33)	1 (6)
Stroke-like episodes	5 (8)	0 (0)	4 (36)	0 (0)	1 (6)
Cognitive dysfunction	5 (8)	1 (3)	3 (27)	0 (0)	1 (6)
Hearing loss	10 (16)	5 (15)	4 (36)	0 (0)	1 (6)
Neuropathy	11 (16)	3 (9)	1 (9)	1 (33)	6 (35)
Sleeping apnea	3 (5)	1 (3)	0 (0)	1 (33)	1 (6)
Ptosis	32 (50)	25 (76)	1 (9)	2 (67)	4 (24)
Ophtalmoplegia	25 (39)	20 (61)	1 (9)	1 (33)	3 (18)
Retinopathy	6 (9)	3 (9)	2 (18)	0 (0)	1 (6)

### Laboratory results

As shown in Table 1, 13 % (N = 8) of the MM patients showed an elevated TnT and 25 % (N = 16) elevated natriuretic peptides at inclusion. Among the 11 CPEO/KSS patients with elevated biomarkers, 10 had elevated NT-proBNP and 1 elevated TnT levels. Six MELAS/-like patients had an increase in biomarkers, TnT elevation being found in 5 and NT-proBNP in 4. CPEO/ KSS and other MM patients showed significantly less frequently TnT elevation when compared to MELAS/-like (p = 0.003). One third (N = 21) of the MM patients had an increase in at least one of the two cardiac biomarkers with no significant difference between groups.

### ECG findings

All patients and controls were in sinus rhythm at presentation. QRS abnormalities were seen in 11 MM patients (left bundle branch block, N = 1; right bundle branch block, N = 5; pathologic Q waves, N = 4 and LV hypertrophy, N = 1) and in two controls (right bundle branch block, N = 1 and left ventricular hypertrophy, N = 1). Similarly, isolated ST/T abnormalities were observed in 11 MM patients (ST-segment depression, N = 1 and T-wave inversion, N = 10) and in two controls (both with T-wave inversion). Five of the CPEO/KSS patients with abnormal ECG showed RBBB and 3 showed T-wave inversions. In the 9 (82 %) MELAS/-like patients with abnormal tracings, the following ECG abnormalities were found: pathologic Q-waves in four, LV hypertrophy in one, LBBB in one and ST/T changes in eight. In total, 18 (28 %) MM patients showed abnormal ECG findings with significantly more MELAS/-like patients presenting with pathological ECGs compared to the other subgroups and to controls.

### CMR findings compared to normal controls

The CMR findings for patients, including the different MM subgroups and controls are listed in Tables 3-4. Compared to controls, the total cohort of MM patients had significantly lower LV end-diastolic volumes (p = 0.028) and increased maximal wall thickness (p = 0.005) with significant more frequent LV hypertrophy (p = 0.016) and higher concentricity as expressed by the ratio of LV mass to end-diastolic volume (p < 0.0001).

**Table 3:** CMR findings (with controls)

	Total MM Patients (N = 64)	CPEO/KSS (N = 33)	MELAS/-like (N = 11)	MERRF (N = 3)	Other MM (N = 17)	Controls (N = 25)	p value
LV end-diastolic volume index ml/m^2^	67 ± 18	61 ± 14*§	85 ± 24	73 ± 15	66 ± 14*	76 ± 17	**0.001**
LV end-systolic volume index, ml/m^2^	25 ± 2	21 ± 8*	37 ± 20	28 ± 10	24 ± 7*	27 ± 8	**0.002**
LV mass index, g/m^2^	56 ± 23	49 ± 11*	90 ± 35	58 ± 17	50 ± 12*	50 ± 12*	**<0.001**
LV ejection fraction, %	66 ± 8	66 ± 7	59 ± 12	62 ± 5	65 ± 6	65 ± 5	0.09
LV ejection fraction <60 %, n (%)	18 (28)	8 (24)	6 (55)	1 (33)	3 (18)	2 (8)	0.07
LV mass/ end-diastolic volume, g/ml	0.84 ± 0.27	0.84 ± 0.24*§	1.07 ± 0.31§	0.71 ± 0.06	0.72 ± 0.22*	0.67 ± 0.11	**<0.001**
Max. wall thickness, mm	10 ± 3	9 ± 2*	14 ± 3§	10 ± 2	10 ± 2*	8 ± 2	**<0.001**
LV hypertrophy in absence of AHT, n (%)	14 (22)	3 (9)*	9 (82)§	1 (33)	1 (6)*	0 (0)*	**<0.001**
RV end-diastolic volume index ml/m^2^	66 ± 14	63 ± 12	69 ± 18	65 ± 9	72 ± 15	72 ± 18	0.21
RV end-systolic volume index, ml/m^2^	30 ± 9	29 ± 9	30 ± 10	32 ± 7	33 ± 10	31 ± 9	0.49
RV ejection fraction, %	55 ± 10	55 ± 11	57 ± 8	51 ± 5	54 ± 10	57 ± 8	0.70
LGE presence, n (%)	21 (33)	12 (36)§	8 (73)§	0 (0)	1 (6)*	1 (4)	**<0.001**
Non-ischemic LGE presence, n (%)	19 (30)	10 (30)§*	8 (73)§	0 (0)	1 (6)*	0 (0)	**<0.001**
Any abnormal CMR findings, n (%)	**34 (53)**	**18 (55)**	**10 (91)**	**1 (33)**	**5 (29)**	**3 (12)**	**<0.001**

*- post Hoc p < 0.05 vs. MELAS/-like; § - vs. Control

LV – left ventricle; RV –right ventricle; AHT – arterial hypertension; LGE – late gadolinium enhancement; CMR – cardiac magnetic resonance

Bold data indicates either significant or most important results

**Table 4:** CMR findings (without controls)

	Total (N = 64)	CPEO/KSS (N = 33)	MELAS/-like (N = 11)	MERRF (N = 3)	Other MM (N = 17)	p value
LV end-diastolic volume index ml/m^2^	67 ± 18	61 ± 14*	85 ± 24	73 ± 15	66 ± 14*	**0.002**
LV end-systolic volume index, ml/m^2^	25 ± 2	21 ± 8	37 ± 20	28 ± 10	24 ± 7	**0.003**
LV mass index, g/m^2^	56 ± 23	49 ± 11*	90 ± 35	58 ± 17	50 ± 12*	**<0.001**
LV ejection fraction, %	66 ± 8	66 ± 7	59 ± 12	62 ± 5	65 ± 6	0.09
LV ejection fraction <60 %, n (%)	18 (28)	8 (24)	6 (55)	1 (33)	3 (18)	0.26
LV mass/ end-diastolic volume, g/ml	0.84 ± 0.27	0.84 ± 0.24	1.07 ± 0.31	0.71 ± 0.06	0.72 ± 0.22*	**0.008**
LV hypertrophy in absence of AHT, n (%)	14 (22)	3 (9)*	9 (82)	1 (33)	1 (6)*	**<0.001**
RV end-diastolic volume index ml/m^2^	66 ± 14	63 ± 12	69 ± 18	65 ± 9	72 ± 15	0.22
RV end-systolic volume index, ml/m^2^	30 ± 9	29 ± 9	30 ± 10	32 ± 7	33 ± 10	0.39
RV ejection fraction, %	55 ± 10	55 ± 11	57 ± 8	51 ± 5	54 ± 10	0.75
LGE presence, n (%)	21 (33)	12 (36)	8 (73)	0 (0)	1 (6)*	**0.001**
Non-ischemic LGE presence, n (%)	19 (30)	10 (30)*	8 (73)	0 (0)	1 (6)*	**<0.001**
Any abnormal CMR findings, n (%)	**34 (53)**	18 (55)*	**10 (91)**	**1 (33)**	5 (29)*	**0.007**

*- post Hoc p < 0.05 vs. MELAS/-like

Bold data indicates either significant or most important results

For the subgroup analysis, when compared to controls (Table 3), CPEO/KSS patients showed significantly lower LV end-diastolic volumes and increased LV concentricity without overt hypertrophy. On the other hand, in relation to controls, MELAS/-like patients presented with increased frequency of concentric hypertrophy with significantly larger wall thickness and LV mass to LV end-diastolic volume index. No significant differences regarding functional parameters and hypertrophy were depicted for MERRF and other MM when compared to controls.

Regarding LGE presence, 21 (33 %) of the MM patients showed LGE in at least one myocardial segment, of which 19 (30 %) patients had non-ischemic patterns (16 intramural and two subepicardial) and two patients (both with CPEO) showed ischemic (subendocardial) LGE. Two patients with predominantly intramural LGE showed also limited transmural extension. Among controls, one patient showed ischemic and none non-ischemic LGE. Compared to controls, non-ischemic LGE was more frequently depicted in MM patients (p = 0.001). This significant difference was primarily due to the higher LGE prevalence in MELAS/-like (N = 8, 73 %) and secondly in CPEO/KSS (N = 10, 30 %) patients, but not in the other two subgroups (MERRF and other MM).

Altogether, 34 (53 %) MM patients presented at least one pathological CMR finding compared to only 3 (12 %) control patients (p < 0.0001). MELAS/-like patients had a pathological CMR in 91 % (N = 10) and CPEO/KSS patients in 55 % (N = 18) of cases, respectively – which was significantly higher compared to the controls. As illustrated in Table 5, cardiac involvement was most frequently diagnosed by a comprehensive CMR study comprising cine- and LGE-images (in 53 %) and rather infrequently based on ECG or cardiac biomarkers, respectively (in 28 % and 33 %, p = 0.008).

**Table 5:** Clinical vs. laboratory vs. imaging findings

	Total MM Patients (N = 64)	CPEO/KSS (N = 33)	MELAS/-like (N = 11)	MERRF (N = 3)	Other MM (N = 17)	Controls (N = 25)	p value
Any (possibly) cardiac symptoms	31 (48)	17 (52)	2 (18)	3 (100)	9 (53)	-	
Elevated TnT or NT-proBNP	21 (33)	11 (33)	6 (55)	1 (33)	3 (18)	-	0.227
Any abnormal ECG findings	18 (28)	8 (24)*	9 (82)§	1 (33)*	0 (0)*	2 (8)	**<0.001**
LV ejection fraction <60 %, n (%)	18 (28)	8 (24)	6 (55)	1 (33)	3 (18)	2 (8)	0.072
LGE presence, n (%)	21 (33)	12 (36)§	8 (73)§	0 (0)	1 (6)*	1 (6)	**<0.001**
Any abnormal CMR findings, n (%)	**34 (53)**	**18 (55)§**	**10 (91)§**	**1 (33)**	**5 (29)**	**3 (12)**	**<0.001**

*- post Hoc p < 0.05 vs. MELAS/-like; § - vs. Control

Bold data indicates either significant or most important results

### CMR pattern according to MM syndrome and relationship to other diagnostic tools

#### CPEO/KSS

**Fig. 1:**
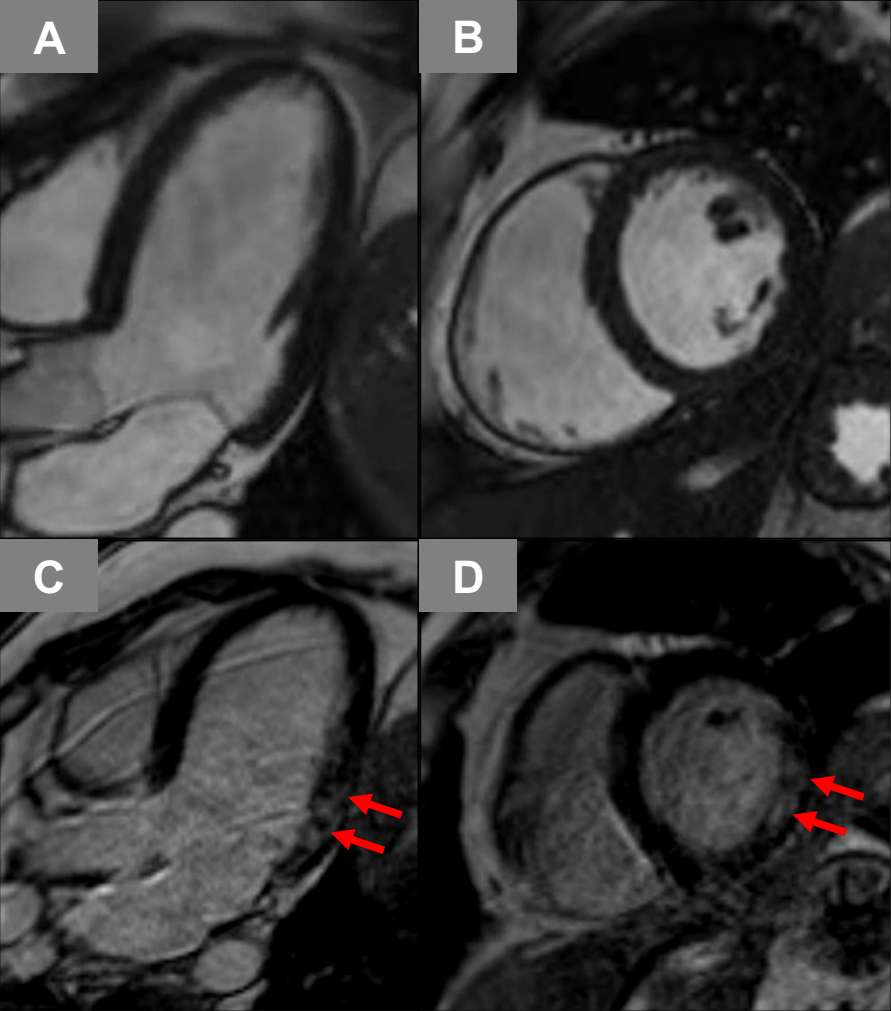
Cine-CMR images in long-axis (**a**) and short-axis views (**b**) with corresponding late gadolinium enhancement (LGE) images (**c-d**). A diffuse intramural pattern of LGE is seen in the basal inferolateral wall segments of the left ventricle (red arrows)

A predominantly intramural pattern of enhancement was noted in 8/10 (80 %) CPEO/KSS patients with proof of non-ischemic LGE (basal inferolateral distribution in six and basal to midventricular septal pattern in two; Fig. 1). A subepicardial pattern was found in the remaining two with basal inferolateral location in both. The three patients with LV hypertrophy in the absence of arterial hypertension or other causal reasons presented only isolated mild septal hypertrophy. There was no relationship between non-ischemic LGE presence or an abnormal CMR finding and an abnormal ECG (p = 0.67 and p = 0.23, respectively). Moreover, there was no significant difference in the occurrence of elevated cardiac biomarkers between patients with (N = 18) and without (N = 15) abnormal CMR findings (29 % vs. 38 %, p = 0.72) among CPEO/KSS patients.

#### MELAS/-like

**Fig. 2:**
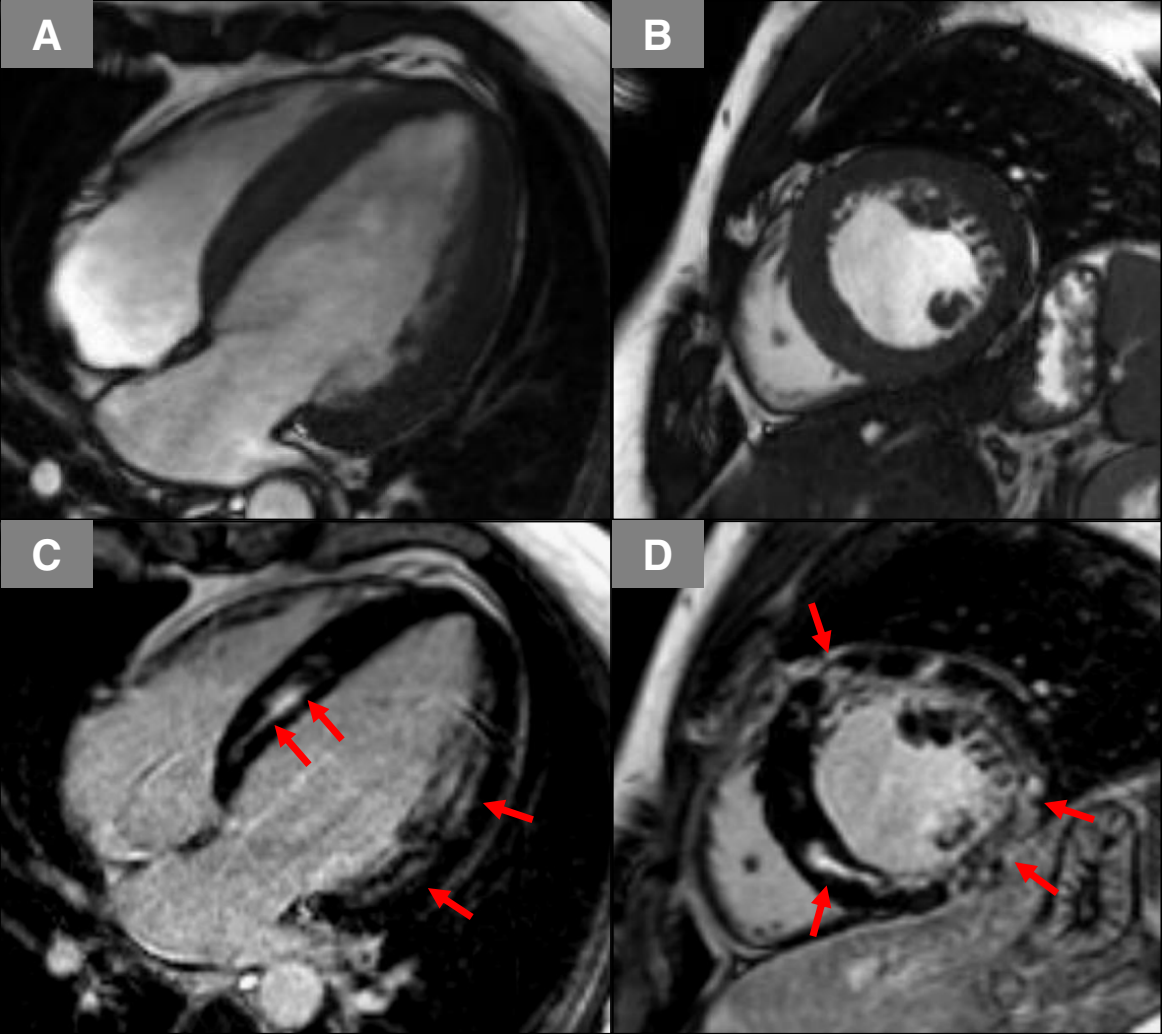
Cine-CMR images in long-axis (**a**) and short-axis views (**b**) with corresponding late gadolinium enhancement (LGE) images (**c-d**). A diffuse, non-ischemic, patchy pattern of LGE is seen in almost all segments of the left ventricle (red arrows)

In comparison to CPEO/KSS, MELAS/-like patients showed significantly larger LV mass with increased LV end-diastolic volumes. Left ventricular hypertrophy was also more frequently encountered in this subgroup compared to CPEO/KSS. Particularly, a concentric pattern was noted in six out of nine (67 %) patients with LV hypertrophy. In addition, MELAS/-like patients showed higher rates for non-ischemic (predominantly intramural) LGE compared to CPEO/KSS. Moreover, a more heterogeneous distribution and extent of LGE, potentially appearing in any of the myocardial segments and occupying between one and 16 segments was observed in MELAS/-like patients (Fig. 2). Furthermore, a significant difference in the presence of an abnormal ECG between patients with (N = 8) and without (N = 3) non-ischemic LGE (100 % vs. 33 %, p = 0.050) could be noted. Moreover, the majority of patients (6/10) with abnormal CMR findings presented with elevated cardiac biomarkers; the only MELAS patient with a normal CMR showed no increase in biomarkers.

#### MERRF

Among the three MERRF patients only one showed pathological CMR findings with a mildly impaired LV ejection fraction and mild septal hypertrophy, however, without presence of LGE. The same patient presented a RBBB and mild TnT elevation.

#### Other MM

As shown in Table 3, among patients with other MM forms (N = 17) only one showed presence of LGE (intramural pattern, location basal inferolateral) associated with mild septal hypertrophy. This LGE-positive patient also demonstrated a mild TnT elevation. Additionally, a mildly impaired LV ejection fraction was found in three patients, in two as an isolated finding and in one associated with mild septal hypertrophy. Another patient showed only isolated mild septal hypertrophy.

## Discussion

Our study presents cross-sectional CMR data in a large cohort of adult patients with MM. We evaluated the presence and pattern of cardiomyopathy and could show that: (1) cardiac abnormalities are frequent among MM patients (53 %) with a non-ischemic LGE pattern being the most frequently encountered finding; (2) at least two different patterns of cardiac involvement can be distinguished, one in CPEO/KSS patients with intramural LGE in the basal inferolateral wall and a second one in MELAS/-like patients with overt concentric hypertrophy and intramural, diffusely located LGE, (3) in both CPEO/KSS and MELAS/-like patients CMR was superior in diagnosing cardiomyopathy compared to ECG and cardiac biomarkers, with a particular diagnostic benefit in CPEO/KSS patients.

### CMR abnormalities

Even though MM-related cardiomyopathy was described in a series of reports, to the best of our knowledge the current work is the first to show that, by means of CMR, structural and/or functional abnormalities are present in the majority of MM patients [4, 6, 9, 10, 13, 14, 16–18]. Moreover, the present study is the first that consistently demonstrates the presence of a concentric hypertrophic remodelling pattern in MELAS/-like patients (and a tendency in CPEO/KSS patients) [9, 17]. This remodelling pattern resembles the myocardial changes found in other inherited systemic disorders not associated with respiratory chain dysfunction such as Friedreich’s ataxia [21–24]. In Friedreich’s ataxia, such a remodelling pattern is generally considered to occur secondary to adaptive changes in response to an impaired cellular energy metabolism which is caused by mitochondrial structural abnormalities [7, 25, 26]. In MELAS, for example, histological examinations of endomyocardial biopsy specimens showed mitochondrial alterations with enlargement and accumulation and, secondarily, disperse interstitial fibrosis and partial myocardial disarray [7, 13, 15]. Considering that one third of MM patients presented with non-ischemic LGE in the present study, CMR represents a new possibility to detect myocardial tissue damage in vivo beyond the known features of LV remodelling and functional abnormalities in this population [13–15, 18, 27].

### CMR-based phenotype patterns in different MM syndromes

Whereas in the few patients with MERRF and in those with other MM no particular CMR changes were found, two potentially characteristic patterns were depicted in CPEO/KSS and MELAS/-like patients, respectively. First, a tendency towards concentric remodelling was noted in CPEO/KSS and second, an overt concentric hypertrophy in addition to LV systolic dysfunction (in half of the cases) was observed in MELAS/-like patients. At first view, these differences could be simply the consequence of a more severe cardiac involvement in MELAS/-like syndrome. Yet, interestingly, despite the known degree of neuromuscular phenotype overlap between syndromes, we found no overlap in cardiac pattern between the two syndromes [1]. Moreover, this difference becomes more prominent when looking at myocardial damage - as depicted by LGE-imaging.

As already reported previously, CPEO/KSS patients tend to show intramural LGE in the inferolateral wall that might either be due to an unequal and predominant distribution of mitochondrial energy deficiency in this area and/or due to an increased mechanical stress in the inferolateral wall [18]. In contrast, MELAS/-like patients do not only frequently show the presence of LGE, but the pattern of LGE is also completely different (Figs. 1-2): strong intramural LGE with focal (and rather patchy) accentuation extending in some cases over all myocardial segments. Besides replacement fibrosis secondary to perturbations in cell energy metabolism with consecutive cell death, another interesting mechanism might be involved in this unusual pattern of LGE seen in MELAS patients (Fig. 2): Similar to the pathogenesis of stroke like cerebral lesions, a hallmark of the MELAS syndrome, a mitochondrial angiopathy with consecutive vasogenic edema and fluid extravasation could be the mechanism for LGE formation in the myocardium [11, 13, 16, 28]. This theory is further supported by the observed presence of myocardial edema as well as perfusion defects in areas with LGE in some of these patients - but not in LGE-positive areas from CPEO/KSS patients [13, 14].

Interestingly, despite the data suggesting a higher prevalence of arterial hypertension in MELAS as adjunctive cause of hypertrophy, none of our MELAS/-like patients had elevated blood pressure [29]. A clear explanation of the exact pathophysiology of these two patterns cannot be given and is beyond the purpose of this paper, but our findings demonstrate that despite the variability in phenotype within each of the two syndromes, relatively specific patterns of cardiomyopathy can be depicted for both.

### Additional value of CMR in the cardiac work-up

First, CMR was superior to the ECG as well as to the measurement of cardiac biomarkers regarding the detection of (signs of) cardiomyopathy for both the whole study group and individual subgroups. Although ECG abnormalities are quite common and are sometimes seen early in the MM disease course, they are mostly unspecific [4, 8, 30]. For example, in a mixed MM population including CPEO and MELAS, Limongelli et al. described ECG changes in 68 % of cases ranging from pre-excitation to conduction abnormalities and ST/T changes [4]. Similarly, in our MM population ECG abnormalities were multifaceted. Moreover, they did not relate to the functional and/or structural CMR findings in CPEO/KSS and found only a weak relationship in MELAS/-like patients. Particularly in CPEO/KSS, approximately one third of the patients with abnormal CMR findings showed normal ECG and negative biomarkers.

Second, even though echocardiography was not routinely performed in the current study, we consider that CMR has a clear advantage over echocardiography for the diagnosis of cardiomyopathy in these patients: The superiority of CMR comes mainly from its ability to sensitively and reproducibly detect structural tissue changes with the depiction of subtle myocardial damage by LGE-imaging [18, 31]. Notably, in 15 % of the CPEO/KSS patients LGE was the only pathological CMR finding. Further, as shown by Pfeffer et al. in a follow-up study conducted in CPEO patients with an initial normal cardiac work-up, only one of the fifteen patients developed new ECG and/or echocardiographic abnormalities over five years making the detection of cardiomyopathy by these techniques even less probable [10]. Therefore, we suggest that a CMR study should be part of the diagnostic approach of cardiomyopathy in MM patients. Early detection of cardiac disease is a prerequisite for the implementation of successful therapeutic strategies and appropriate risk stratification with timing of follow-up studies. In this context, the recently published ESC guidelines for the management of hypertrophic cardiomyopathy also address mitochondrial diseases such as MELAS and suggest to perform a CMR study at initial presentation if local expertise in this technique is available [32]. We advocate that CMR should be considered in all patients with MM at their baseline assessment – if local resources and expertise permit it. Moreover, we suggest that CMR follow-up studies in MM patients with pathological CMR results (particularly with presence of LGE and/or impaired systolic function) and potentially progressive disease – particularly those with MELAS and MELAS-like disease - should be considered every 6-12 months while a follow-up study every 4-5 years will be sufficient in (adult) patients with no involvement of the heart muscle.

In MM patients with a rather hypertrophic pattern of cardiomyopathy (e.g. MELAS or MELAS-like patients), ß-blockers, angiotensin-converting enzyme (ACE) inhibitors and angiotensin receptor blockers may potentially help to (at least) slow down the progression of cardiomyopathy – as was shown in aetiologically different forms of HCM [32]. In this context, it should be emphasized that MM such as MELAS and MELAS-like diseases are mentioned as specific HCM forms in the recently published “2014 ESC guidelines on diagnosis and management of HCM”.

CMR may not only help to accurately assess the thickness of the LV wall, but the mere presence of LGE in e.g. MELAS patients may precede the occurrence of LV hypertrophy and thereby used as a diagnostic tool for a timely initiation of the aforementioned treatment options – prior to the occurrence of LV hypertrophy and advanced myocardial scarring. Our own (unpublished preliminary) experiences suggest that cardiac disease progression regarding myocardial scarring occurs surprisingly fast in some patients with MELAS or MELAS-like diseases. Therefore, the most accurate diagnostic tools available should be used in order to detect the first signs of cardiomyopathy.

Paralleling other genetic cardiomyopathies, e.g. hypertrophic cardiomyopathy, non-ischemic LGE detection could play an additional prognostic role in the risk stratification of MM, particularly in MELAS patients [33–35]. It has been already shown that MELAS patients have a high incidence of cardiac death as well as arrhythmia and heart failure events, primarily related to LV hypertrophy [2, 3]. Since our preliminary experiences in MELAS/-like patients point to a rather disproportionately extensive pattern of LGE compared to the degree of LV hypertrophy (Fig. 1), one may even hypothesize that detection of LGE in these patients could have a superior prognostic value than in other non-MM diseases that are associated with LV hypertrophy. However, this issue is a clinically relevant topic for future research and so far, there are no follow-up data involving LGE-CMR in MM patients.

### Limitations

The first limitation is the small number of patients, particularly suffering from MERRF. This fact obviously limits us to draw appropriate conclusions regarding presence, pattern and clinical value of cardiomyopathy in these patients. A recent study from Wahbi et al. found cardiac abnormalities in 44 % of the 18 MERRF patients included comprising LV dilatation or hypertrophy with or without systolic dysfunction [12]. A direct comparison to our data is difficult as this study used echocardiography and other definitions for LV hypertrophy and dilatation.

A second limitation is the absence of histopathological data. The patients did not fulfil the current guideline recommendations for performing EMB and therefore, this procedure was rarely performed [36].

A third limitation lies in the fact that controls were free from cardiac symptoms while MM patients were symptomatic at inclusion. Nevertheless, in MM patients cardiac symptoms are often unspecific and rather difficult to differentiate from the coexisting neuromuscular disease. Moreover, no association between symptoms and CMR findings was found.

## Conclusion

Cardiac involvement is a frequent finding in MM patients – and particularly present in KSS/CPEO as well as MELAS/-like patients. Despite a high variability in clinical presentation, CPEO/KSS patients typically show an intramural pattern of LGE in the basal inferolateral wall whereas MELAS patients are characterized by overt concentric hypertrophy and a rather unique, focally accentuated and diffusely distributed LGE.

## Additional file

Additional file 1: Table S1. Detailed overview of underlying genetic mutations.

## Abbreviations

BNP: Brain natriuretic-peptide
CK: Creatine kinase
CMR: Cardiovascular magnetic resonance
CPEO: **C**hronic **p**rogressive **e**xternal **o**phthalmoplegia
DCM: Dilative cardiomyopathy
LGE: Late-gadolinium-enhancement
LV: Left ventricle
LV-EDV: Left ventricular end-diastolic volume
LV-ESV: Left ventricular end-systolic volume
LV-EF: Left ventricular ejection fraction
KSS: **K**earns-**S**ayre **s**yndrome
MELAS: **M**itochondrial **e**ncephalopathy with **l**actic **a**cidosis and **s**troke-like episodes
MERRF: **M**yoclonic **e**pilepsy with **r**agged-**r**ed **f**ibers
MM: Mitochondrial myopathy
RV: Right ventricle
RV-EDV: Right ventricular end-diastolic volume
SSFP: Steady-state-free-precession
TnT: Troponin T
