# Supplementary material for: Characteristic cardiac phenotypes are detected by cardiovascular magnetic resonance in patients with different clinical phenotypes and genotypes of mitochondrial myopathy
Source: J Cardiovasc Magn Reson. 2015 May 22;17(1):40. doi: 10.1186/s12968-015-0145-x (PMC4490728; doi:10.1186/s12968-015-0145-x)
Supplement: Additional file 1: Table S1. — Detailed overview of underlying genetic mutations. [file 12968_2015_145_MOESM1_ESM.doc]

**Supplemental table: Detailed overview of underlying genetic mutations**

| **Patient ID** | **Phenotype** | **Result of molecular genetic analysis from blood** |
| --- | --- | --- |
| 3 | MELAS | MTTL1, m.3243A>G |
| 5 | Other | mt-tRNA, 5591G>A |
| 8 | CPEO | MTTL1, m.3243A>G |
| 10 | Other | AMPD 1, C34-T mutation |
| 12 | CPEO | C214+13 G>A |
| 17 | MERRF | MTTK, m.8344A>G |
| 20 | MELAS | MTTL1, m.3243A>G‡ |
| 27 | Other | mtDNA, 11778G>A point mutation |
| 29 | CPEO | mtDNA, (1399 G>A) A467T mutation |
| 31 | CPEO | mtDNA, C489A mutation |
| 41 | MELAS-like | mt-tRNA, m.4295 A>G† |
| 42 | MELAS-like | mt-tRNA, m.4295 A>G † |
| 43 | MELAS-like | mt-tRNA, m.4295 A>G † |
| 44 | MELAS-like | mt-tRNA, m.4295 A>G † |
| 45 | Other | Repeat expansion in one allele to (GCG)9 in the PABPN1 gene |
| 46 | MELAS | MTTL1, m.3243A>G‡ |
| 50 | MERRF | MTTK, m.8344A>G |
| 59 | MELAS | MTTL1, m.3243A>G |
| 63 | MELAS | MTTL1, m.3243A>G |
| 64 | MELAS-like | MRPL44, (c*.*467T>G, p.Leu156Arg) |

† - members of the same family
